# Integrated care for frail elderly compared to usual care: a study protocol of a quasi-experiment on the effects on the frail elderly, their caregivers, health professionals and health care costs

**DOI:** 10.1186/1471-2318-13-31

**Published:** 2013-04-12

**Authors:** Isabelle Natalina Fabbricotti, Benjamin Janse, Wilhelmina Mijntje Looman, Ruben de Kuijper, Jeroen David Hendrikus van Wijngaarden, Auktje Reiffers

**Affiliations:** 1 Erasmus University Rotterdam, Institute of Health Policy and Management, P.O. Box 1738, Rotterdam, DR, 3000, The Netherlands; 2 Erasmus University Rotterdam, Institute of Health Policy and Management, P.O. Box 1738, Rotterdam, DR, 3000, The Netherlands; 3 Erasmus University Rotterdam, Institute of Health Policy and Management, P.O. Box 1738, Rotterdam, DR, 3000, The Netherlands; 4 Huisartsenpraktijk Arnemuiden, Prins Bernhardstraat 2, Arnemuiden, EZ, 4341, The Netherlands; 5 Erasmus University Rotterdam, Institute of Health Policy and Management, P.O. Box 1738, Rotterdam, DR, 3000, The Netherlands; 6 Walcherse Huisartsen Coöperatie, Adriaen Coortestraat 61, Middelburg, DM, 4336, The Netherlands

**Keywords:** Integrated care, Frailty, Vulnerable elderly, Prevention, Primary care, Quasi-experimental design, Effectiveness elderly care, Caregiver, Health professionals

## Abstract

**Background:**

Frail elderly persons living at home are at risk for mental, psychological, and physical deterioration. These problems often remain undetected. If care is given, it lacks the quality and continuity required for their multiple and changing problems. The aim of this project is to improve the quality and efficacy of care given to frail elderly living independently by implementing and evaluating a preventive integrated care model for the frail elderly.

**Methods/design:**

The design is quasi-experimental. Effects will be measured by conducting a before and after study with control group. The experimental group will consist of 220 elderly of 8 GPs (General Practitioners) who will provide care according to the integrated model (The Walcheren Integrated Care Model). The control group will consist of 220 elderly of 6 GPs who will give care as usual. The study will include an evaluation of process and outcome measures for the frail elderly, their caregivers and health professionals as well as a cost-effectiveness analysis. A concurrent mixed methods design will be used. The study population will consist of elderly 75 years or older who live independently and score a 4 or higher on the Groningen Frailty Indicator, their caregivers and health professionals. Data will be collected prospectively at three points in time: T0, T1 (3 months after inclusion), and T2 (12 months after inclusion). Similarities between the two groups and changes over time will be assessed with t-tests and chi-square tests. For each measure regression analyses will be performed with the T2-score as the dependent variable and the T0-score, the research group and demographic variables as independent variables.

**Discussion:**

A potential obstacle for this study will be the willingness of the elderly and their caregivers to participate. To increase willingness, the request to participate will be sent via the elders’ own GP. Interviewers will be from their local region and gifts will be given. A successful implementation of the integrated model is also necessary. The involved parties are members of a steering group and have contractually committed themselves to the project.

**Trial registration:**

Current Controlled Trials ISRCTN05748494

## Background

With an aging population, caring for the increasing number of the frail elderly is a challenge for the Dutch healthcare system [1,2]. The frail elderly are those with a disease or infirmity associated with advanced age, which is manifested by demonstrable mental, psychological, emotional or physical dysfunction to the extent that the person is incapable of adequately providing for his or her own health and personal care presently or in the near future [3,4]. In 2010, 16% (2.6 million) of the Dutch population was 65 years or older, of which 10% was 75 years or older and 7% was 80 years or older [5]. Of the elderly population in 2010, 25% were considered frail. As a result of reduced mortality rates and the demographic shift, there will be a higher frail population in need of long-term care in the near future. The percentage of the frail elderly is estimated to increase to 68% in 2030 [6]. In the meantime, the demand for services already strains the professional workforce and caregiver burden [7-9].

The frail elderly are an important group within the elderly population because their diminished compensation capacities make them, their caregivers, and society most able to benefit from changes in social and healthcare arrangements [10,11]. Due to their complex and continuously changing health and social problems, the frail elderly need a wide range of services over a long period of time [12]. However, the reluctance of the frail elderly to report their growing impairments to their doctors impedes interventions at a stage when preventive care could diminish further mental, psychological or physical deterioration [13]. Approximately 30% of the Dutch frail elderly receive no domestic, personal, home or private care [14]. They solely rely on their own judgment or that of their caregivers for seeking help or for performing their daily activities. Timely recognition of unmet needs can avoid crisis situations or the overburdening of the caregiver. It can also improve social wellbeing [15-17].

Changes also occur in the attitudes of the elderly toward care. These changes also necessitate changes in the organization of care. The frail elderly no longer silently accept the care that they are given and now demand their care meets their needs. Patient-centeredness has become a legitimating base for healthcare provision and has been reinforced by laws that strengthen patient's rights. These laws also force providers to provide the care that the elderly want and need at the right time and place [5,18-20]. A supply-oriented approach and the fragmentation in the organization of the elderly care today inhibit progress on this issue. Service is still often characterized by a lack of continuity and coordination on the behalf of involved providers. Responsibility for the whole continuum of care is absent and results in inefficient and ineffective care [21,22]. The specific needs of the frail elderly and their caregivers, budget restraints and patient-centered views call for new and more effective organizational structures.

The integration of health services and social services for the frail elderly has gained tremendous attention as a means to accomplish this. There is a widespread belief that the integration of these will enhance satisfaction, quality of life, efficiency, and health outcomes and will also decrease costs [23-26]. The rationale behind this stems from the fact that a single service provider is usually unable to respond to all the needs. This prohibits efficiency in the delivery process. To meet the multiple needs of the frail elderly in an efficient and effective manner, some claim that numerous service providers will need to combine their efforts in a coordinated manner [27-29]. There is also mounting evidence that confirms beliefs that the development of integrated care arrangements can be cost effective and enhance quality [30-38].

Though widely acknowledged and pursued, the implementation and evaluation of integrated services for the frail elderly has not yet reached its full potential. Much is still unknown regarding how services can be integrated and the effects of integration. In this study, a new integrated model for the frail elderly, the Walcheren Integrated Care Model, will be developed and evaluated. Walcheren refers to the region in the Netherlands where the study takes place. The Walcheren Integrated Care Model is in accordance with scientific evidence and addresses the design elements that affect the quality of care. It has an umbrella organizational structure involving case management, multidisciplinary teams, protocols, consultations, and patient files. It will be an organized provider network with evidence-based needs assessments [29,32,33]. All elements are embedded in the model. However, more types of health professionals participate in the model than other studies have previously investigated. General practitioners, geriatricians, home health care workers, paramedics, social workers, pharmacists, and mental health care professionals all take part in the designed model. In contrast with other models, this model also contains a preventive element: a screening tool to detect frailty in the elderly. Finally, the model is being evaluated on a broader range to obtain a comprehensive evaluation and determine possible trade-offs between effects.

This article describes the study design of the evaluation of the Walcheren Integrated Care Model compared with traditional care. The development and evaluation of the model are part of the National Care for the Elderly Program (NPO), which is funded by the Netherlands Organization for Health Research and Development (ZonMW; project number 313030201).

### The intervention: the Walcheren integrated care model

**Figure 1:**
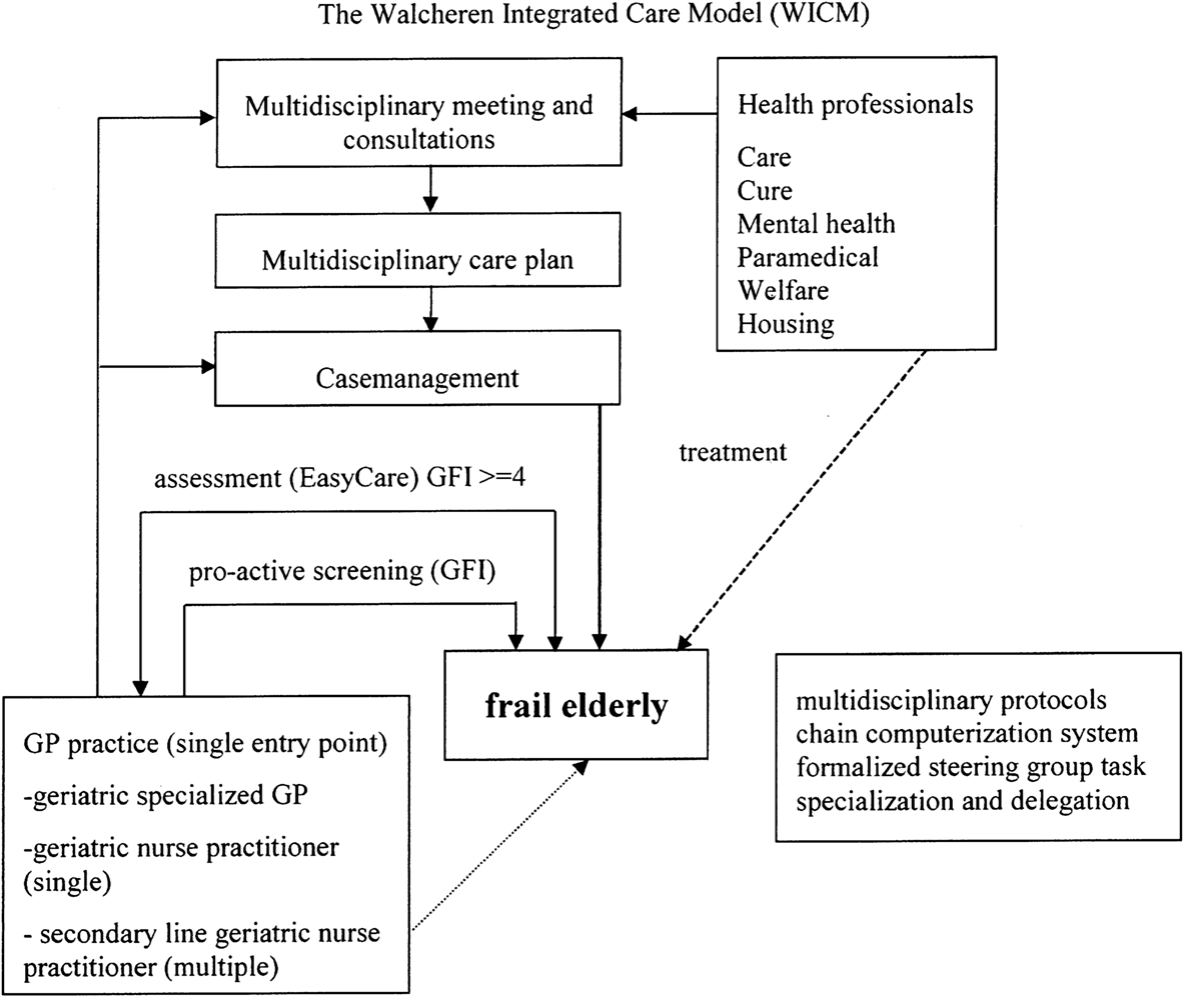
The Walcheren Integrated Care Model (WICM).

The Walcheren Integrated Care Model (WICM) is a comprehensive integrated model for the detection and assessment of needs and the assignment and evaluation of care for independently living frail elderly. The model comprises ten elements: a screening tool for the detection of frailty in the elderly, a single entry point, an evidence-based comprehensive need assessment tool, a multidisciplinary individualized service plan, case management, multidisciplinary team consultation and meetings, protocol-led care assignment, a steering group, task specialization and delegation, and a chain computerization system (see Figure 1).

The frail elderly aged 75+ years are identified by their general practitioner (GP) by the Groningen Frailty Indicator (GFI), a tool for the detection of frailty. The GFI is a 15-item questionnaire that measures decreases in physical, cognitive, social, and psychological functioning. Scores can range from 0 to 15 [39,40]. A geriatric nurse practitioner that works at the GP practice sends the GFI questionnaire to the homes of the elderly and then contacts them by telephone if they do not respond. When necessary, elderly are helped at home to complete the questionnaire. A geriatric nurse practitioner and GP calculate the GFI score. Elderly with a GFI ≥4 are identified as frail and assigned to a case manager. The geriatric nurse practitioner is the case manager for elderly with single needs. A secondary line geriatric nursing specialist is assigned as case manager if the needs are multiple or of a complex nature.

The case manager then sets up a meeting with the elderly to assess their needs with the EASYcare instrument. EASYcare is an evidence-based comprehensive need assessment instrument that assesses (instrumental) activities of daily life, cognition, and mood. It also contains a module for converting care requirements relating to welfare, residence, and care into treatment goals [41]. The goals are drawn up in consultation with the elderly and their caregivers. Explicit attention is paid to the necessary support and guidance of the caregivers. The results of the assessment are described by the case manager in an individualized care plan. The case manager also creates a proposal for required care and care objectives.

The proposed plan is then discussed in a multidisciplinary meeting led by the GP. Depending on treatment goals, the meeting is also attended by other health professionals who may be needed. During the meeting, a multidisciplinary care plan will be approved, actions and care paths will be discussed, and agreements will be made about the care to be deployed and the activities of all persons involved. The treatment plans of each professional are included in the care plan. The GP harmonizes the care plan with the elderly and their caregiver and obtains permission for its implementation. A chain computerization system accessible by the health professionals involved will be used for the multidisciplinary care plan. The professionals will automatically receive an email in the event of changes in use of care or a transfer.

The case manager is responsible for admittance to the required services, the planning and coordination of care delivery, and periodical evaluation of the care plan. Thus, the case manager arranges obligatory need assessment, monitors the elderly at least every six months for one year, and supports the multidisciplinary team by arranging meetings and streamlining the necessary exchange of information. The responsibilities and activities of the involved professionals and case manager are formalized in agreed protocols with predefined modes of referral and collaboration. During the process, the GP practice functions as a single entry point. It is the gate through which elderly and professionals can access the expertise and services of all health and social care professionals and organizations. The GP and case manager work in close collaboration to ensure timely and correct care assessment and provision. To be able to fulfill their tasks, the GPs must have completed an executive training in geriatric care, a course in GP consults and EASYcare training. The case managers must have successfully attended the EASYcare training and a course in case management.

## Methods and design

### Aim

The aim of the project is to improve the quality and efficacy of care given to frail elderly living independently by their caregivers and health professionals. It seeks to do this by implementing, evaluating, and disseminating an integral care model for the frail elderly. Living independently is defined as living at home or in a sheltered accommodation without receiving other forms of integrated care. The research questions for the evaluation study is as follows: What are the effects of the Walcheren Integrated Care Model on the caregivers, health professionals, the organization of care and the healthcare costs for the frail elderly, and what are the effects on the quality and efficacy of the care given to the frail elderly living independently?

### Study design

The study has a quasi-experimental design in which the effects will be measured before and after the study. A control group will also be used. The study includes an evaluation of process and outcome measures for the frail elderly, their caregivers, and health professionals, as well as a cost-effectiveness analysis. To evaluate the effects, a combination of qualitative and quantitative research methods will be used. (See Tables 1, 2, 3 and 4). The study protocol has been reviewed by the medical ethics committee of the Erasmus Medical Centre, Rotterdam, the Netherlands, under protocol number MEC-2013-058. They waived further examination as the rules laid down in the Medical Research Involving Human Subjects Act did not apply.

**Table 1:** Outcome measures and data collection frail elderly

**Outcome and instrument**	**Method**	**Data collection time**		
		**T0**	**T1**	**T2**
*Primary outcomes*				
**Quality of life**				
ICECAP	Interview elderly	x	x	x
EQ-6d	Interview elderly	x	x	x
SF-36	Interview elderly	x	x	x
Cantril’s self-anchoring ladder	Interview elderly	x	x	x
*Secondary outcomes*				
**Perceived health**				
SF-36	Interview elderly	x	x	x
**Social functioning**				
SF-36	Interview elderly	x	x	x
**Mental well being**				
SF-36	Interview elderly	x	x	x
**Physical functioning**				
KATZ-15	Interview elderly	x	x	x
**Health care use**				
Self-reported	Interview elderly	x	x	x
Reported by GP	File research	x	x	x

**Table 2:** Outcome measures and data collection caregivers

**Outcome and instrument**	**Method**	**Data collection time**		
		**T0**	**T1**	**T2**
**Perceived health**				
SF-36	Interview caregiver or mailed questionnaire	x	x	x
**Objective burden**				
Short version iBMG	Interview caregiver or mailed questionnaire	x	x	x
instrument objective burden informal care				
**Subjective burden**				
Carer-Qol	Interview caregiver or mailed questionnaire	x	x	x
SRB	Interview caregiver or mailed questionnaire	x	x	x
CSI+	Interview caregiver or mailed questionnaire	x		x
Perseverance time	Interview caregiver or mailed questionnaire	x		x
ASIS	Interview caregiver or mailed questionnaire	x		x
**Quality of life**				
SF-36	Interview caregiver or mailed questionnaire	x	x	x
Cantril’s self-anchoring ladder	Interview caregiver or mailed questionnaire	x	x	x
**Use of community services**				
Self-reported	Interview caregiver or mailed questionnaire	x	x	x
CSAI	Interview caregiver or mailed questionnaire	x		x

**Table 3:** Outcome measures and data collection health professionals

**Outcome and instrument**	**Method**	**Data collection time**		
		**T0**	**T1**	**T2**
**Knowledge**				
Self-constructed VAS	Mailed questionnaire	x		x
**Job satisfaction**				
Job satisfaction scale	Mailed questionnaire	x		x
**Subjective burden**				
SRB	Mailed questionnaire	x		x
**Objective burden**				
Self-reported by elder	Interview elderly	x	x	x
Self-reported by professional	Time tracking form	x	x	x
Reported by GP	File research	x	x	x

**Table 4:** Process measures and data collection

**Outcome and instrument**	**Method**	**Data collection time**		
		**T0**	**T1**	**T2**
**Degree of integration**				
Self-constructed questionnaire	Mailed questionnaire	x		x
**Satisfaction health professionals**				
Self-constructed questionnaire	Mailed questionnaire	x		x
Relational coordination Survey	Mailed questionnaire	x		x
Self-reported satisfaction	Diaries	x	x	
	Interviews			x
	Focus groups	x	x	x
**Satisfaction frail elderly**				
CQ-index	Interview elderly	x	x	x
Self-constructed questionnaire	Interview elderly	x	x	x
**Satisfaction caregiver**				
CQ-index	Interview caregiver or mailed questionnaire	x	x	x
Self-constructed questionnaire	Interview caregiver or mailed questionnaire	x	x	x

### Power calculation

We will include 220 elderly in both the experimental and control group. We expect a 10% loss to follow-up (due to mortality, re-housing, impossibility or unwillingness to participate further) between inclusion and T1 and a 20% loss between T1 and T2. The sample is sufficient to detect changes in our primary measure of quality of life. Assuming an average effect size of 0.5 and significance of 5%, this gives a power of 0.997. If we assume a small effect size of 0.3 with a significance of 5%, this still supplies sufficient power at 0.837. Interfering variables will also play a role. At an average effect size (f2) of 0.15 and significance of 5%, assuming five independent variables, the power is 0.97. Even with 15 independent variables, the power remains sufficient at 0.856.

### Study sample: sampling and eligibility criteria

Sampling will take place at GP practices in Walcheren. The experimental group will consist of the elderly patients of 8 GPs from 3 GP practices located in the east of Walcheren who will provide care according to the WICM. The control group will consist of 6 GPs from 5 GP practices in the north, south, and west of Walcheren who will provide traditional care. All elderly aged 75+ years in these practices who live independently will be asked to complete the GFI, along with several demographic questions and a consent form. Approximately 900 elderly in both the experimental and control practices will be contacted. The questionnaire is accompanied by a letter from the GP to raise the likelihood of response and assure that the elderly are well informed. After being sent a reminder, the elderly will be contacted by telephone or visited at home to be asked to participate and to help complete the questionnaire if necessary. These activities are expected to result in an 80% response rate. Elderly will be included if they score ≥4 on the GFI, if they have signed the consent form, or if they are able to make that decision themselves. Exclusion criteria are as follows: elderly on a waiting list for a nursing home, elderly who are not able to decide themselves if they want to participate (e.g., in case of dementia), and elderly with a life expectancy of <6 months due to a terminal illness. Included elderly will be asked to provide contact information for their informal caregiver. The caregivers will be contacted either by telephone or face-to-face during the first visit from the researchers at the home of the elderly subjects. They will be asked to fill in a written consent form if they agree to participate. Non-respondents will be contacted again by telephone. A response rate of 60% is expected. Health professionals will be selected based on their function and region of employment. An estimated 400 questionnaires will be sent to health professionals in the experimental and control groups. We expect a response rate of 50%.

### Data collection and instruments: frail elderly

Outcome data and data on demographics (age, sex, living arrangement, education, and marital status) will be collected with questionnaires and file research at three points in time: T0, T1 (3 months after inclusion), and T2 (12 months after inclusion). Research has shown that effects can be expected 3 months after starting to use the EASYcare instrument [41]. The T2 measurement takes place to determine long-term effects. All elderly will be visited at home by trained interviewers recruited from the region of Walcheren to ensure a cultural fit with the elder. Interviewers will have a background in healthcare to ensure a high-quality interview. Every elder will be given a gift at T1 as a token of appreciation and to motivate further participation. File research will occur at the GP practices. The following instruments will be used (see Table 1):

#### Perceived health

##### SF-36

The SF-36 measures eight concepts: physical functioning, bodily pain, role limitations due to physical, personal, and emotional health problems, emotional well-being, social functioning, energy/fatigue, and general health perceptions [42,43]. The items regarding perceived current health and changes in health will be used.

#### Social functioning

##### SF-36

The SF-36 question on social functioning ‘During the past 4 weeks, to what extent has your physical health or emotional problems interfered with your normal social activities with family, friends, neighbors, or groups?’ will be used.

#### Mental wellbeing

##### SF-36

The 5-items scale on emotional wellbeing from the SF-36 will be used.

#### Quality of life

##### ICECAP

The ICECAP instrument was developed for elderly and measures their quality of life using the following 5 dimension on the capacity to perform certain actions and achieve certain states: attachment, security, role, enjoyment, and control. Each dimension consists of one question that can be scored on four levels [44].

##### EQ-6d

The EuroQol (EQ6D) is used to measure quality of life in terms of valued health and is composed of the dimensions mobility, self-care, usual activities, pain/discomfort, anxiety/depression, and cognitive functioning [45,46]. Each dimension is scored on three levels: ‘no problems,’ ‘some problems,’ and ‘severe problems.’ The EQ-6d will also be used to calculate cost-utilities of health care.

##### SF-36

Questions based on the SF-36 on perceived current quality of life and the quality of life compared with one year ago will be used.

##### Cantril’s self-anchoring ladder

Perceived quality of life will be measured with the Cantril’s ladder, a measurement technique that asks subjects to mark their satisfaction with life from 0 to 10 [47].

#### Physical functioning

##### KATZ-15

The Katz-15 will be administered to measure physical functioning by means of 15 yes or no questions covering domains of activities of daily functioning, such as bathing, transferring, eating, and dressing [48,49].

#### Health care use

##### Questions on self-reported use

Use of healthcare will be measured with 16 questions regarding the use of seven domains of care (hospital admissions, unplanned care, respite care, medical, paramedic, psychosocial care, and daycare). Elderly will be asked if they make use of care, and if so, how often (in days or hours depending on the type of care).

##### File research

The files of the elderly from the GPs will be analyzed regarding health care use. Data will be collected on the same domains as described above and compared with self-reported use.

### Data collection and instruments: caregivers

Outcome data and demographic data (e.g., age, sex, income, relationship, and living with loved one) from the caregivers will be collected with questionnaires at three time points: T0, T1 (3 months after inclusion), and T2 (12 months after inclusion). Caregivers will be sent a questionnaire or interviewed at the same time as the elder at their home. Caregivers will also be given a gift at T1. The questionnaire is composed of the following instruments (see Table 2):

#### Perceived health

##### SF-36s

As for the elderly, the items on perceived current health and changes in health from the SF-36 health survey will be used.

#### Objective burden

##### Short version Erasmus iBMG instrument “objective burden informal care

This instrument measures and divides the time spent on the elderly into the following domains: household tasks, personal care, help with moving and contacts with family, friends and health care providers, and medical technical tasks [50]. Caregivers will be asked if they give help, and if so, how many hours per week.

#### Subjective burden

##### Carer‒Qol

The CarerQol will be used to measure the impact of informal care [51,52]. The CarerQol-VAS assesses happiness with a horizontal Visual Analogue Scale (VAS) with 0 (‘completely unhappy’) and 10 (‘completely happy’) as endpoints. The CarerQol-7d describes seven dimensions of burden: fulfillment, support, relational and mental health problems, problems with combining daily activities, finances, and physical health. The answer categories are ‘no’, ‘some’ and ‘a lot of problems.’

##### Self-related burden VAS (SRB)

The SRB will be used to measure the overall perceived burden. The SRB asks how straining the care for the loved one is with a horizontal VAS ranging from 0 (‘not straining at all’) to 10 (‘much too straining’) [53].

##### Caregiver Strain Index+ (CSI+)

The CSI+ will be used to measure perceived strain. The CSI+ is an extended version of the 13-item instrument CSI, which only measures negative dimensions of the caregiver situation. The CSI+ adds 5 items on positive dimensions covering the areas of patient characteristics, subjective perceptions of the care-taking relationship by caregivers, and emotional health of caregivers [54,55].

##### Question on perseverance time

The question of how long the caregiver anticipates being able to pursue his tasks as a caregiver will be asked, with answers ranging from less than two weeks to more than two years [56].

##### Assessment of the informal care situation (ASIS)

To assess the desirability of the caregiving situation, the ASIS will be used, which is a horizontal VAS ranging from 0 (‘worst imaginable caregiving situation’) to 10 (‘best imaginable caregiving situation’) [51].

#### Quality of life

The same *SF-36* based questions and Cantril’s self-anchoring ladder for the elderly will be used.

#### Use of community services

##### Community Service Attitude Inventory (CSAI)

The CSAI is a 25-item Likert-type scale that will be used to measure the attitude and willingness of caregivers toward the use of community services [57].

##### Survey question

Caregivers will be asked if they use community services.

### Data collection and instruments: health professionals

Data on the outcomes will be collected from GPs, nursing home doctors, geriatrists, geriatric nurse practitioners, secondary line geriatric nursing specialists, specialists in hospitals, home care employees, mental health professionals, and paramedical specialties with the following instruments (see Table 3):

#### Knowledge

##### Questionnaire

At the end of the project, a questionnaire will be distributed to the health professionals involved in the experimental and control groups by their organization of employment. This will help ensure the privacy of contact information. The questionnaire is composed of two questions regarding the assessment of the health professional. It assesses his or her knowledge on the frail elderly and his or her knowledge of the roles and tasks of other health professionals involved in the care for the frail elderly. Answers are given for the current situation and the situation 18 months previously and are measured with a VAS ranging from 0 to 10.

#### Job satisfaction

##### Job satisfaction scale

The job satisfaction scale will be part of the questionnaire. This instrument is a 10-item questionnaire with questions on extrinsic and intrinsic job satisfaction [58,59]. Health professionals will be asked to assess how satisfied they are now and 18 months previously on a scale ranging from 1 (‘extremely unsatisfied’) to 7 (‘extremely satisfied’).

#### Subjective burden

##### Self-related burden VAS

Inspired by the SRB, a similar VAS will be used to measure the overall perceived burden. As the SRB was developed for caregivers, the question will be transformed into the question ‘How straining is it to give care to the frail elderly?’ Scoring measures the current situation and the situation 18 months previously with a horizontal VAS ranging from 0 (‘not straining at all’) to 10 (‘much too straining’).

#### Objective burden

##### File research and questionnaire

File research and the questions on healthcare use by the elder as mentioned above will be used to determine the time spent on care. For the time calculation, the volume of care will be multiplied by a mean time determined by consensus with the health professionals (e.g., 40 minutes per house visit by a GP).

##### Time tracking form

The GPs, geriatric nurse practitioner and secondary line geriatric nursing specialist will also keep track of the time spent on managing cases and coordinating tasks, time spent on conferring with health professionals, and time spent on multidisciplinary meetings per elder. A time tracking format will be developed to this end.

### Data collection and instruments: cost-effectiveness

The question that is central to the economic analysis is whether the WICM is cost-effective compared with traditional care. The outcome parameter used is cost per QALY (quality-adjusted life-year). For this, the EuroQol (EQ-6D) will be used to measure the quality of life of the elderly persons and will subsequently be converted into disability-adjusted life-years (DALYs). For the cost calculation, the volume of care will be linked to the actual, integral cost per medical service [60]. This will be used to make the instructions for cost research in economic evaluations [61]. Thus, the total care consumption of the elderly will be determined. The above-mentioned patient files, questionnaire, and time tracking form will provide insight into which care was received per elder, how much and from whom.

### Data collection and instruments: process indicators

To determine the level of coordination, coherence, and satisfaction with care processes, the following process indicators will be measured with questionnaires, file research, interviews, diaries, and focus groups (see Table 4).

#### Degree of integration

##### Questionnaire

To determine the degree of coherence, continuity, and co-operation, a questionnaire will be developed based on a systematic review of integration indicators and instruments for measuring integration. The questions will be part of the questionnaire sent to the health professionals as described above. Health professionals are again asked to assess the current levels of integration and those 18 months previously.

#### Health professionals’ experiences with the quality and process of care

##### Questionnaire

Questions on satisfaction with the process of care and level of integration will be derived from the above-mentioned results of the systematic review.

##### Relational coordination survey for patient care

The quality of the relationships and communications between health professionals will be measured with the relational coordination survey for patient care, an instrument covering the following dimensions: shared goals, knowledge and respect, frequency and timing of communication, and problem-solving orientation of the communication [62,63].

##### Diaries

The geriatric nurse practitioner and secondary line geriatric nursing specialist will be asked to keep a diary of their experience with the WICM. Every 3 months, a researcher will briefly interview the geriatric nurses over the telephone to discuss their experiences based on the diary.

##### Interviews

After the completion of the experiment, interviews will be held with involved professionals. Discussions will cover their experience with the WICM, conducive and non-conducive factors that played a role and any adjustments that the model may require.

##### Focus groups

For both the experimental and control regions, 3 focus groups will be organized for the health professionals and patient organizations involved. These focus groups will be used to gain insight into satisfaction with the model and its integration. The groups will also strengthen the analysis by reflecting on the results of the study.

The frail elderly and caregiver experiences with the quality and process of care.

##### Consumer quality index (CQ-index)

The CQ-index, a Dutch standardized method for measuring experiences of patients/clients with health care, will be used. Covered domains are quality of the health professionals, information, participation, treatment, communication, and received care [64,65]. CQ-questionnaires are developed for different types of care. The CQ-questionnaire for home care will be used as a reference point and be completed with questions on the coherence and coordination of care. Elderly will be asked at T0, T1, and T2 regarding their experience of the care and care processes. Caregivers will be asked at T0, T1, and T2 regarding their experiences of the care given to their elder and the care and attention that they receive from health professionals.

### Data analysis

The experimental and control groups will be described at every point in time with descriptive statistics. Similarity of characteristics between the two groups will be assessed with t-tests, chi-square tests, and Fisher’s exact tests. Bivariate analyses and regressions with the demographic characteristics will determine multicollinearity and correlations with the process and outcome measures. All analyses will be controlled for differences in baseline characteristics and demographic characteristics. For the self-constructed questionnaires, factor analyses and reliability analyses will be performed to determine construct validity. To determine changes over time, t-tests will be performed for each process and outcome measure. For each measure, regression analyses will be performed with the T2-score as the dependent variable and the T0-score, the research group (experimental or control), and demographic variables as independent variables. With subgroup analyses, potential variation between study results between subgroups will be analyzed.

## Discussion

### Implementation of the model

The development, evaluation, and dissemination of the Walcheren Integrated Care Model depends on its successful implementation. Research has shown that the implementation of integrated care is a very difficult and laborious task [66,67], especially regarding the proposed model because it focuses on integration across the entire continuum of care for all frail elderly. Other developmental strategies mainly focus on small programs for a targeted group or on a small part of the care process [26]. Additionally, when integrated arrangements are being implemented successfully in one setting, one is often unable to achieve dissemination on a wider scale [32]. Furthermore, developing integrated care arrangements is as much a process of social and cultural integration as it is structural integration. The success of implementation is shaped by the interests and cultures of the health professionals and the social relationships between them. Integration involves aligning the work of health professionals and convincing them to work together from a patient-centered viewpoint [29,68]. Several activities are and will be deployed to ensure that these challenges are overcome.

The involved professionals are all represented in a steering group that forms the umbrella under which the model is developed and disseminated. The steering group forms a Joint Governing Board that provides the necessary provider network, which is further strengthened with guidelines and protocol-led agreements. All patient representatives support the project, and the health insurer CZ is supporting the project financially. The basis for collaboration is also laid down in the formalization of agreements on the regional policy and involves integrated care for all elderly: the so-called ‘structured care of the elderly module.’ The project follows from these structures and will be able to make use of them.

Though administratively secure, the project will eventually be affected by the willingness of the partners to review tasks and delegate and accept new responsibilities thrust upon them. Acceptance of the role of a GP as coordinator is an essential aspect of this. GPs cannot claim this coordinating role for themselves. It will have to be given to them based on the confidence of all ‘players’ and by an agreement that a coordinating role for the GP is a suitable mechanism for improving the care for the frail elderly. A basis for this has already been established. The Walcheren GP Co-operation Care Group, the GP Co-operation in Veere, a working group of elderly patients and various partners in the region have agreed, within the recommendations and preconditions of the National Association for GPs (NHG), that creating a single entry point from the GP practices is the point of departure for setting up structured care of the elderly in Walcheren.

The feasibility of the experiment will also be enhanced by knowledge obtained in the region regarding instruments and collaboration that includes the elderly. Knowledge about using the GFI instrument was obtained during a pilot with the GFI instrument among elderly persons aged 85+ years. Consultations with elderly patients aged 65+ years have already started in three practices. Due to the broad involvement and experiences of health professionals, no major obstacles are expected regarding the model implementation. The pressures on providing care may increase for GPs because the use of the GFI instrument will provide them with information about the frail elderly who were previously unknown. This additional work pressure will be calculated in advance to prepare the GPs for the workload. The extra burden on GPs in the control region is particularly related to time registration and participation in interviews. These extra efforts will also be discussed with them in advance.

Embedding the experiment in other projects is essential over the long term. The experiment does not stand alone. A dementia care-chain and CVA care-chain are also being developed in Walcheren. The protocols developed will guarantee the link with the EASYcare instrument as used in this experiment. The steering group will ensure coherence between the various projects. The GPs in this project are also involved with developing the dementia care-chain. Their personal involvement in both projects will guarantee harmonization.

### Evaluation study

The choice for a quasi-experimental design instead of a randomized control trial may seem suboptimal to some. However, in many studies on organizational change, randomization is impractical, impossible or even undesirable [69]. This is the case in our study as health professionals cannot give traditional care and care according to the model at the same time. Blinding is impossible. For the elderly, it is undesirable to receive care from a different GP or organization from one previously used.

However, choosing for a quasi-experimental design presents our study with some challenges. The absence of randomization makes results subject to contamination by confounding variables [70]. Potentially confounding variables have been accurately defined based on literature, experiences of health professionals and comparable studies. Inclusion and exclusion criteria are set. However, there is no guarantee that some confounding variables will be missed. It is also conceivable that differences found in the experimental group are not the result of the intervention but of the additional attention given by both health professionals and interviewers [71]. It is debatable if this “Hawthorne-effect” is really problematic because increased and patient-centered attention for the frail elderly is one of the goals of the model. Irrespective of the design chosen, the biggest potential obstacle is the willingness of the elderly and their caregivers to participate in this study over the longer term. To increase willingness, a request to participate will be sent, as described above, via the elders’ own GPs, interviewers will be from the region and gifts will be given.

## Abbreviations

WICM: Walcheren Integrated Care Model; GP: General practitioner; GFI: Groningen Frailty Indicator; SF-36: Short form (36) health survey; ICECAP: Index of capability for older people; EQ-6d: EuroQol (6 dimensions); SRB: Self-related burden; VAS: Visual analogue scale; CSI: Caregiver strain index; ASIS: Assessment of the Informal Care Situation; CSAI: Community Service Attitude Inventory; iBMG: Institute of Health Policy and Management; Qaly: Quality adjusted life years; Dalys: Disability adjusted life years; CQ-index: Consumer quality index.

## Competing interests

The authors declare that they have no competing interests.

## Authors’ contributions

AR and RK were responsible for the design of the Walcheren Integrated Model and the commitment of all involved parties. IF was responsible for the development of the evaluation study. IF, AR, RK and JW drafted the first version of the proposal. BJ and WL completed the proposed instruments. AR, project leader for the implementation of the model and IF, project leader for the evaluation study, wrote the final proposal and were responsible for obtaining funding for the study. All authors read and approved the final manuscript.

## Pre-publication history

The pre-publication history for this paper can be accessed here:

http://www.biomedcentral.com/1471-2318/13/31/prepub
